# Magnetic Resonance Imaging Study on Blebs Morphology of Ahmed Valves

**DOI:** 10.5005/jp-journals-10008-1174

**Published:** 2015-01-15

**Authors:** Joana Ferreira, Fernando Fernandes, Madalena Patricio, Ana Brás, Cristina Rios, Ingeborg Stalmans, Luis Abegão Pinto

**Affiliations:** Consultant, Department of Ophthalmology, Lisbon’s Hospitals, Central Lisbon, Portugal; Consultant, Department of Ophthalmology, Lisbon’s Hospitals, Central Lisbon, Portugal; Consultant, Department of Neuroradiology, Lisbon’s Hospitals, Central Lisbon, Portugal; Consultant, Department of Neuroradiology, Lisbon’s Hospitals, Central Lisbon, Portugal; Consultant, Department of Neuroradiology, Lisbon’s Hospitals, Central Lisbon, Portugal; Professor, Department of Ophthalmology, University Hospitals, Leuven Belgium; Consultant, Department of Ophthalmology, Lisbon’s Hospitals, Central Lisbon, Portugal

**Keywords:** Glaucoma, Intraocular pressure, Bleb morphology, Ahmed valve, Magnetic resonance imaging.

## Abstract

**Purpose:** To determine the morphometric parameters of filtration blebs of a valved aqueous humor drainage device.

**Materials and methods:** Orbital magnetic resonances imaging (MRI) was taken after implantation of an Ahmed valve (FP7 model). Outcomes of the analysis were intraocular pressure (IOP) and the bleb’s morphometric analysis (volume, height, major and minor axis). Associations between IOP and the imaging-related study variables were explored by Spearman’s correlation test.

**Results:** Eleven patients underwent orbital MRI examination. Recordings were taken after a mean of 2.7 months (1-6 months) after surgery. IOP was significantly lower than its preoperative values (17.6 ± 6.4 mm Hg *vs* 36.1 ± 6.4 mm Hg, p < 0.01). Mean bleb volume was 856.9 ± 261 mm^3^ and its height, major and minor axis were 5.77 ± 1.9, 14.8 ± 2.9 and 8.14 ± 3.6 mm, respectively. A positive correlation was detected between IOP and mean height (r = 0.77, p = 0.048) and major axis (r = 0.83, p = 0.03). Interestingly, the overall bleb volume was related to IOP levels immediately prior to surgery (r = 0.75, p < 0.01). Additionally, the posterior part of the plate was found to be displaced from the scleral surface in five cases (45%).

**Conclusion:** Ahmed valve’s bleb morphology seems to correlate with both the pre- and postoperative IOP, which might suggest a clinical benefit of administering aqueous suppressants pre- as well as postoperatively. The plate of the device may show a significant dislocation from its initial surgical implantation site.

**How to cite this article:** Ferreira J, Fernandes F, Patricio M, Brás A, Rios C, Stalmans I, Pinto LA. Magnetic Resonance Imaging Study on Blebs Morphology of Ahmed Valves. J Curr Glaucoma Pract 2015;9(1):1-5.

## INTRODUCTION

Implantation of aqueous humor drainage devices (ADD) is an increasingly popular surgical option in glaucoma patients with noncontrolled intraocular pressure (IOP).^1-3^ These implants establish a drainage route for the aqueous humor from the anterior chamber into a subconjuctival reservoir.^4,5^ The normal functioning of this structure (known as filtering bleb) is of paramount importance for the surgical success of this surgery.^6-8^ However, unlike blebs seen in other filtering surgeries (such as trabeculectomies), these ADD reservoirs are not fully seen at slit-lamp due to their more posterior location. Accordingly, the standard morphological criteria for assessing a filtering bleb, such as vascularization pattern,^9^ thickness of the Tenon’s capsule^10^ and the ‘ring of steel’ formation^11^ are of limited value since the biggest portion of the bleb remains unseen. One way to study this bleb morphology is through the use of imaging technology, such as magnetic resonance imaging (MRI). This noninvasive technology allows the characterization of the several orbital structures while still providing a high image resolution.^12-14^ However, the two studies where MRI has been used for this purposes refer either to a pediatric population or to a study mixing small-sized samples from two different ADD (Ahmed valve and Baerveldt tube). As these caveats precluded a wider generalization of the results, a study on an adult population in which only a single type of ADD was implanted. Accordingly, the purpose of this study was to determine the clinical correlations of MRI-bleb imaging data from glaucoma patients implanted with an Ahmed valve.

## MATERIALS AND METHODS

### Subject Groups

Patients with recent, uncomplicated implantation of an ADD (Ahmed Valve FP7 model, New World Medical Inc, Rancho Cucamonga, CA) on the superior temporal quadrant for an uncontrolled IOP were asked to participate in this prospective study. Inclusion criteria were the following: older than 18 years old, willing to participate in the study and having had uncomplicated surgery between 1 and 6 months before the study. Patients were excluded if possessing a connective tissue disease or orbital-related pathology (such as Graves’ ophthalmopathy), having had a combined ADD implant procedure with cataract surgery, a background of previous ophthalmologic surgeries involving the manipulation of the orbit or extra-ocular muscles (e.g. strabismus, scleral buckle) or any postoperative complications (atalamia, tube erosion, plate extrusion) requiring surgical reintervention. Another exclusion criterion was the existence of contraindications to the magnetic resonance (e.g. pacemaker implant).

### Experimental Design

This prospective study was conducted at the Glaucoma and Neuroradiology Department of Centro Hospita-lar de Lisboa Central. This study was approved by the Local Ethics Committee and was conducted in accordance with the good clinical practice within the tents of the declaration of Helsinki. Each patient was required to sign an informed consent statement before enrolling in the study and prior to any study measurement or examination being made. At study visit, a complete ophthalmology observation was performed, which included visual acuity, IOP measurement by Goldman applanation tonometry, slit-lamp observation and fundoscopy with a 78D lens. Data from medical records detailing indication of drainage device implantation, pre-surgical IOP and current ocular hypotensive therapeutics were collected. For ethical reasons, patients were not discontinued from their regular medical therapies (IOP-related or not).

### Surgical Technique

All surgeries were performed by the same surgeon (LAP), using the same technique. In brief, the surgical technique involved a fornix-based conjunctival flap in the superior temporal quadrant. The tube was primed using balanced salt solution with a 30 gauge needle. The plate was secured to the sclera with its anterior edge 8 to 10 mm posterior to the limbus using non-absorbable sutures (8.0 nylon®). A sclera tunnel was created starting approximately 2 mm from the limbus and, using a 23 gauge needle, the tunnel was advanced into the anterior chamber of the eye. The tube was trimmed bevel up and inserted through the tunnel into the anterior chamber aiming toward the center of the pupil. The tube was secured to the underlying sclera by two sutures with non-absorbable material (10.0 nylon®). A donor scleral or corneal patch was placed over the scleral surface to cover most of the tube’s path. This was sutured to the sclera with 10.0 nylon® sutures. The conjunctiva was reapposed using absorbable sutures (7.0 vicryl®). Finally, a subtenon methylprednisolone 40 mg/ml (Depo-medrol®) injection was performed. Postoperative treatment was a fixed combination of tobramycin and dexamethasone (Tobradex^®^) four times daily for 4 weeks.

### Magnetic Resonance Imaging

Imaging data acquisition was performed in a MRI device of a Tesla (Harmony®, Siemens Medical Solutions, Erlangen, Germany) using a surface coil for optimization of image capture on the same day as the study visit. Each patient was submitted to an orbital high-resolution MRI, with T1 and T2 weighted images. Axial and coronal images were captured with 2 mm of thickness using a 256 × 256 matrix over a visualization field of 210 × 210 mm. The Digital imaging and communications in medicine (DICOM) images were analyzed recurring to the AW Volumeshare ® 5 (General Electric Healthcare, Little Chalfont, United Kingdom) image processor. Images with presence of artefacts were excluded. All images were interpreted by the same observer (CR), masked to the patients’ ophthalmological data. The Ahmed valve plate was identified as a low density circumlinear structure in both images T1 and T2, placed adjacent to sclera in the eyeball superotemporal quadrant. The filtration bleb, identified with a collection of humor signalled as water (high intensity on T2 images and low intensity on T1 images), was quantified using T2 images. The volumetric calculations were made using the sum of structure areas identified as filtration bleb in each coronal section where it was detected and multiplied by the thickness of each section, such as described in the literature.^12^

### Statistical Analysis

The correlations between the analysed variables were performed recurring to the Spearman correlation. Values of p < 0.05 were considered statistically significant (Graphpad Prism® ver. 5.0; Graphpad Software Inc, CA). The descriptive results are presented as the mean ± standard deviation.

## RESULTS

Table 1 summarizes the clinical and demographic information of patients included in the study. Eleven patients were included (6 males), with a mean age of 61.6 ± 15 years old. Tube surgery was chosen due to their specific medical history: corneal transplant (3 patients), neovascular glaucoma secondary to central retinal vein occlusion (4 patients), history of previously failed trabeculecto-mies (2 patients) and uveitic glaucoma (1 patient). IOP was significantly reduced from preoperative values of 36.1 ± 6.4 mm Hg to 17.6 ± 6.4 mm Hg at the study visit (p < 0.01). No patient was still under topic steroid therapy. Only one patient was under topical IOP-lowering therapy (latanoprost 0.05% qd + timolol 0.5% bid).

### Filtration Bleb Imaging

**Figs 1A to C:**
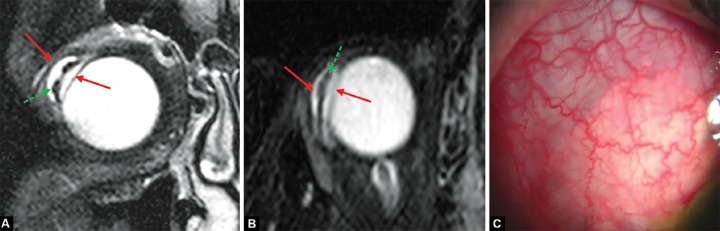
Example of an Ahmed valve imaging implanted in a right eye: (A and B) Depict a T2-weighted coronal and axial MRI image of a device implanted in the temporal superior quadrant. The device is the low intensity () segment (green arrow) between the two high intensity, liquid components (red arrows) of the bleb and the vitreous. This illustrates a nondirect contact between the posterior part of the plate and the scleral surface, (C) shows the same quadrant as observed from slit-lamp biomicroscopy

The coronal sectional images analysis in T2 identified a filtration bleb mean height of 5.77 ± 1.89 mm (measured in the major distance between two parallel plans at the scleral surface and at the external surface of the hyperin-tensive bleb component). Bleb’s major axis (i.e. in sagittal direction), was 14.8 ± 2.89. Minor axis (i.e. in coronal direction) was 8.14 ± 3.59 mm. The bleb volumetric calculus registered a mean value of 859.6 ± 261 mm^3^ (Table 2). Interestingly, the posterior part of the plate was found to be displaced from the scleral surface in five cases. Figure 1 shows the device and filtration bleb images by MRI and slit-lamp biomicroscopy.

**Table 1:** **Table 1:** Clinical characteristic of the study patients

*Patient*		*Age* *(years)*		*Gender*		*Pre-op IOP* *(mm Hg)*		*Current IOP* *(mm Hg)*		*Time elapsed* *(months)*		*Ocular comorbilities*	
1		32		M		43		35*		2		Uveitic glaucoma	
2		55		M		36		20		2		Penetrating keratoplasty	
3		53		M		40		12		6		Penetrating keratoplasty	
4		79		M		35		20		5		Previous failed trabeculectomies	
5		71		F		30		14		3		Retinal detachment (PPV)	
6		85		F		44		16		1		Neovascular glaucoma	
7		65		M		35		11		2		Neovascular glaucoma	
8		65		F		43		18		1		Penetrating keratoplasty	
9		68		F		37		16		3		Previous failed trabeculectomies	
10		58		F		23		14		2		Neovascular glaucoma	
11		47		M		31		18		3		Neovascular glaucoma	
Mean		61.6 (15)		―		36.1 (6.4)		17.6 (6.4)		2.7 (1.5)		―	

*Patient 1 was under topical IOP-lowering medication (prostaglandin analog and nonselective beta-blocker). Pre-op: Preoperative; IOP: Intraocular pressure; PPV: Pars plana vitrectomy; M: Male; F: Female

### Correlations between Imaging and Clinical Variables

**Fig. 2:**
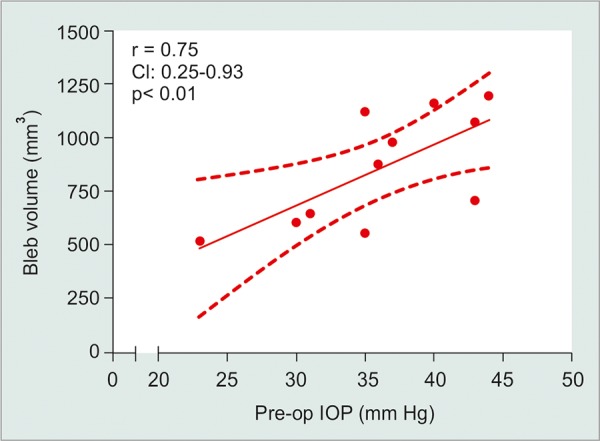
Correlation between bleb volume and preoperative intraocular pressure (IOP). A positive correlation is seen between these two parameters: r = 0.75 (CI 0.25-0.93), p < 0.01. Statistics performed using Spearman’s correlation test

Bleb’s height and major axis showed a positive correlation with postoperative IOP (r = 0.77, r = 0.83; both p < 0.05). Interestingly, the bleb’s overall volume correlated with the IOP levels immediately prior to surgery (r = 0.75; p < 0.01; Fig. 2). Neither age nor time lapse correlated with any of the imaging variables (p > 0.05 in all comparisons).

**Table 2:** **Table 2:** Volume and morphometric measurements of the filtering bleb

*Volume*		*Vertical axis*		*Major axis*		*Minor axis*	
856.9		5.77		14.8		8.14	
261 mm^3^		1.89 mm		2.89 mm		3.59 mm	

Data presented as mean (SD)

## DISCUSSION

Ahmed valve’s bleb morphology seems to have a particular relationship with pre- as well as postoperative IOP. In the current study, a higher preoperative IOP was associated with a higher overall bleb volume. Our results support the hypothesis that an *ab initio* large pressure gradient could lead to a major distension of the subcon-junctival reservoir. As in any type of distension, this bleb increase can continue until a balance is reached between the pressure acting over the capsule wall and its resistance. In filtering surgery, a mechanical stress is a known factor stimulating a number of inflammatory pathways that leads to collagen deposition and increased wall thickness.^15,16^ This thickened wall provides increased resistance to being crossed by the aqueous humor may thus result in an IOP increase.^17,18^ As the aqueous humor (AH) content of inflammatory cytokines is related to the IOP level, then at least initially, the subconjunctival space of high-IOP patients would be bathed by AH with higher concentrations of inflammatory cytokines.^6^ This might be an explanation as to why valved implants are associated with greater thickness of the capsular wall^19^ and more probability of bleb dysmorphia and creation of giant reservoirs,^20,21^ as nonvalved surgery is usually done resorting to techniques that prevent, at least in an early stage, a free drainage of aqueous humor. In this sense, this information would further reinforce the recent notion that early AH suppression may be of value in Ahmed valve implantation. A corollary of this assumption would also admit that a more aggressive decrease in preoperative IOP (with either mannitol iv or acetazola-mide *per os)* could have decreased bleb volume.^22^

In addition to the bleb volumetry, its overall configuration may also be important for its IOP-lowering efficacy, as Laplace’s law shows that the pressure acting on a wall would depends on the radius of its circumference. Accordingly, a reduction in the diameter of the capsule would reduce the mechanical stress on the capsule wall and thus the need to reinforce its collagen content.^18^ Reversely, larger bleb dimensions would be associated with a larger radius of circumference and increased wall stress, with subsequent thickened wall and higher resistance to AH reabsortion. In keeping with this concept, our data indeed show that longer, higher blebs are associated with higher IOP values.

One particular intriguing observation in our study was the luxation of the plate from their original implant site in a significant proportion of our patients. Indeed, in five patients (out of 11), the low-intensity plate was no longer placed over the sclera, but rather separated from it by a liquid layer. This plate movement―undetected at the slit-lamp―may explain why tube position inside the anterior chamber may change over time (as the posterior end of the tube may have shifted from its original location). Further studies would be needed to verify the clinical relevance of this plate luxation.

One limitation of our study is its small sample size. This is, however, the largest study on this subject, outpacing the previous study (n = 8). Moreover, the latter study split its numbers between two different ADD (Ahmed valves, n = 4 and Baerveld tubes, n = 4). As bleb formation may be different between various ADD models, generalization and overall comparison of the results between these studies should be done carefully. Furthermore, recruited was limited to uneventful surgeries and postoperatives. Adding in such small series a number of factors that could interfere with the healing process (such as combined cataract surgery or need for revision surgery) would have introduced a significant bias in this pilot study. Data from those more complicated surgeries would, however, be of great interest in clarifying the mechanisms behind ADD wound healing and further studies will be needed. Another limitation is the short follow-up. Of note, most patients had the MRI scans around 3 months, and the low IOP values suggest the majority of patients were no longer going through the hypertensive phase usually seen in these implants. As success rates from these ADD decline over time, it would have been interesting to perform the same analysis in patients with a longer follow-up and assess their bleb’s characteristics.

## CONCLUSION

MRI is a non-invasive technique that allows the evaluation of the posteriorly-located ADD bleb. Ahmed valve’s bleb geometry is correlated with its IOP-lowering efficacy, and overall bleb volume is related preoperative IOP levels. The plate of the device may show a significant dislocation from its initial surgical implantation site.
